# Parents' Attitude Towards Their Feverish Children in Buraydah, Saudi Arabia

**DOI:** 10.7759/cureus.61000

**Published:** 2024-05-24

**Authors:** Omar E Alomari, Omer Alyahya

**Affiliations:** 1 Family Medicine, Qassim Health Cluster, Buraydah, SAU; 2 Family Medicine, Family Medicine Academy, Buraydah, SAU

**Keywords:** saudi arabia, health seeking behavior, practice, knowledge, fever, parents

## Abstract

Background: Fever is a very common problem among pediatric age groups globally. Parents' adequate knowledge and practice make a huge difference in the areas of cost as well as time. The objective of this study is to determine parents' knowledge and practice about their feverish child and the socio-demographic characteristics associated with such knowledge and practice.

Methods: A cross-sectional study was conducted among 194 parents attending primary healthcare centers (PHCs) by using a convenient sampling method. Informed consent was obtained from each participant. Data were collected through a validated self-administered questionnaire and later analyzed with SPSS software. For inferential statistics, the chi-square test was applied.

Results: Of the 194 participants in our study, 59.8% were men (n=116) and 40.2% were women (n=78). About 37.1% (n=72) of parents had defined the maximum normal temperature for children as 37.5°C. Additionally, 71.6% (n=139) of the parents were concerned about convulsions in feverish children. Approximately 39.2% (n=76) of parents considered PHC doctors to be their source of information. About 70.1% (n=136) of participants applied cold compresses when their child developed a fever. Nearly 88.1% (n=171) of parents preferred to visit the doctor when their child had a high fever. There was a statically significant association observed between occupation categories and source of information (P<0.05).

Conclusion: Based on the study results, it was found that parents had poor knowledge about defining the normal body temperature. Approximately two-thirds of the study participants had good practices about health-seeking behavior.

## Introduction

Fever is one of the most common complaints, resulting from an increase in the hypothalamic set point. It is the reason for health visits in about 70% of the pediatric age group [1]. Although it can cause uncommon severe complications in children, such as febrile seizures, it is generally viewed as a manageable condition that can be treated at home using non-pharmacological or pharmacological remedies that can be purchased without a prescription [2]. Parents often view fever as a disease rather than a symptom or indication of illness [3]. As a result, they may feel anxious when their child has a fever and struggle to determine the seriousness of the illness [4].

The term "fever phobia" was coined by Dr. Barton Schmitt in 1980 to refer to the anxious perspective that parents have towards fever [5]. Insufficient understanding among parents about the origins of fever and misunderstandings regarding its impact on their children's health often result in excessive psychological distress, anxiety, and fear [6]. Taken together, these factors can increase healthcare expenses and promote the unwarranted use of antibiotics. Moreover, parents may make excessive phone calls and visits to physicians, potentially leading to unnecessary laboratory tests and even prescribing unnecessary medication to their children solely to appease their own concerns [7,8].

A study conducted in 1997 investigated the viewpoints and beliefs of 707 British mothers regarding fever and its management. The study found that almost 59% of mothers were apprehensive during the febrile episode, while 17% reported being highly anxious [3]. Similarly, a study conducted in Denmark revealed comparable results, with parents displaying misconceptions about fever and expressing concern about temperatures that were considered to be normal [9]. Furthermore, a study conducted in Australia discovered that merely 1.4% of 401 parents correctly assessed and treated fever, while 64.6% were administering incorrect dosages of antipyretics [10].

Additional studies indicate that educational attainment, socioeconomic status, and cultural background are the principal factors that influence parents' knowledge and decision-making regarding childhood fever [5,11]. In a study conducted in Morocco, 264 parents were surveyed about their understanding of fever and how they manage their children who have a fever. The study revealed that merely 3.5% of the participants correctly defined fever [12].

In Saudi Arabia, a study examining parental perceptions of fever in children discovered that over 70% of parents had an inadequate comprehension of fever, including its definition, what constitutes a high fever, and the threshold temperature that requires antipyretics [13].

In prior research conducted by AlAteeq et al. among Saudi parents, the assessment of their knowledge and practices regarding the management of fever at home indicated insufficient knowledge and poor practices. The study also revealed excessive use of non-prescribed fever medication, which could potentially lead to the wastage of healthcare resources [14]. Furthermore, a study in Saudi Arabia revealed that, in terms of the definition of high fever, 67% of parents provided the correct response. It is noteworthy that only 3% of parents could accurately identify the best site for measuring body temperature [15].

Studies conducted in other countries have demonstrated inadequate knowledge and misconceptions among parents concerning childhood fever. However, there is a scarcity of research on this topic in Saudi Arabia [13,14].

This study was conducted to investigate parents' awareness of childhood fever management. The study aimed to identify the level of awareness and factors that influence parental behavior concerning fever management practices. Our belief was that obtaining an accurate assessment of parental knowledge and practices regarding childhood fever would enable us to develop and implement educational programs tailored to the needs of the region, aimed at promoting rational fever management and reducing the burden on healthcare spending.

## Materials and methods

Target population and study setting

Our study target population was parents who had children <14 years of age residing in Buraydah city, and the study was conducted among primary healthcare center (PHC) attendees. Table 1 presents the inclusion and exclusion criteria.

**Table 1:** The inclusion criteria and exclusion criteria.

Inclusion criteria	Exclusion criteria
People who included in this study are parents of children aged <14 years and parents aged from 18 years to 65 years	Parents with mental illness
	Non-cooperative parents

Study design

A cross-sectional study was conducted to assess the parents' knowledge and practice about their feverish child during the period from March 2023 to March 2024.

Questionnaire tool development

The questionnaire was adapted from a previous study conducted in Riyadh, after reviewing the literature and obtaining approval from the principal investigator of the Riyadh study to use the same questionnaire [14]. We also adjusted some questions in the tool.

Our questionnaire included demographic, knowledge, and practice variables. These variables were included in the following order: age, gender, educational level of parents, income, number of children, normal temperature and high fever, concerns about fever, sources used by parents to diagnose and manage fever, action taken by parents when their child is feverish, whether they usually measure temperature, what medication they give to reduce fever, if there are any difficulties with its use, and health-seeking behavior of parents towards their feverish child. A validated, structured, self-administered pretested questionnaire and some semi-structured variables were used to collect the data. As per the World Health Organization (WHO), fever can be defined as a body temperature more than 38°C.

Sample size calculation

Based on the OpenEpi sample size calculator, the following parameters were used to estimate the sample requirement in our study, which was 384: a confidence level of 95%, a margin of error of 5%, an anticipated frequency of 50%, a design effect of 1.0, and the population size of Qassim was 1.3 million, according to the general authority for statistics of Saudi Arabia in 2022.

As our research was conducted in Buraydah, the capital city of Qassim province, which represents 50% of the total Qassim population, we took 50% (384/2=192) of the total sample estimate. The final sample in our study was 192. However, I collected 194 samples.

Sampling strategy

We included four PHCs in Buraydah, which are located in the north, south, east, and west parts of Buraydah. The required sample was equally divided among four PHCs, and therefore, approximately 48 parents were recruited from each of the selected PHCs to meet our eligibility criteria. For the participant’s selection, we distributed the self-administered questionnaire to the parents at the waiting hall by convenience sampling method.

Ethical considerations

Ethical committee approval was obtained from the Qassim Regional Bioethics Committee, with approval number 607-44-16300; then, we started collecting the data from the selected PHCs. Informed consent was obtained from all participants, and confidentiality was maintained as the names and IDs of participants were not included.

Data analysis

The data were analyzed using IBM SPSS Statistics for Windows, Version 21.0 (Released 2012; IBM Corp, Armonk, New York, United States). This included descriptive analysis and frequencies and proportions of categorical variables, while the mean and standard deviations were calculated for continuous variables. For the categorical variable analysis, the chi-square test was applied.

Pilot study

The pilot study was conducted among 10 participants to determine the feasibility, order of questions, and field difficulties. The pilot study sample was not included in the main study sample.

## Results

Out of 194 study participants, 82% (n=155) were above the age of 35 and 59.8% (n=116) were males. The response rate in the study population was 84.3% (194/230). The mean number of children among participants’ parents was 2.22 and the range of the number of children is 6. About 69.6% (n=135) of parents stated that fever is harmful and 29.4% (n=57) were mentioned very harmful. Regarding parent’s knowledge about the maximum normal temperature for children, 37.1% (n=72) considered 37.5°C as normal. Over 50% of parents (52.6%, n=102) perceived their child as having a fever when their child's temperature reach 38°C.

Table 2 revealed that about 38.7% (n=75) of participants were from other occupation (the majority were police and military). Regarding the education level of the participants, 62.4% (n=121) of mothers and 72.7% (n=141) of fathers had completed college-level education. Almost two-thirds of the study population (62.4%, n=121) had an income level of more than 9000 SR per month. 

**Table 2:** Demographic characteristics of the study population (n=194).

Variable	Number	Percentage
Age group (years)		
18-25	1	0.5
26-35	34	17.5
36-45	91	46.9
46-55	64	33.0
56-65	4	2.1
Gender		
Male	116	59.8
Female	78	40.2
Occupation		
Teacher	62	32.0
Housewife	57	29.4
Others (police, military,...)	75	38.7
Father educational level		
Primary	4	2.1
Secondary	4	2.1
High school	37	19.1
College	141	72.7
Postgraduate	8	4.1
Mother educational level		
Primary	8	4.1
Secondary	7	3.6
High school	54	27.8
College	121	62.4
Postgraduate	4	2.1
Income of family		
1000-3000 SR	2	1.0
3001-5000 SR	20	10.3
5001-7000 SR	12	6.2
7001-9000 SR	39	20.1
>9000 SR	121	62.4

Table 3 depicts that, regarding knowledge towards feverish children, about 87.1% (n=169) of parents used forehead hand touch to measure the temperature. Only 41.2% (n=80) of parents brought their child to a PHC or ER visit when their child had a fever.

**Table 3:** Attitude status of parents towards feverish children in the study population. PHC: primary healthcare center.

Attitude	Mostly (%)	Sometimes (%)	Never (%)	Total (%)
Forehead hand touch	169 (87.1)	19 (9.8)	6 (3.1)	194 (100)
Oral measure	50 (25.8)	47 (24.2)	97 (50)	194 (100)
Auditory measure	70 (36.1)	28 (14.4)	96 (49.5)	194 (100)
Axillary measure	35 (18)	28 (14.4)	131 (67.5)	194 (100)
Rectal measure	17 (8.8)	4 (2.1)	173 (89.2)	194 (100)
PHC or ER visit	80 (41.2)	80 (41.2)	34 (17.5)	194 (100)

Table 4 states that the majority (71.6%, n=139) of the parents were concerned about convulsions, and the second concern was dehydration at about 20.6% (n=40). Regarding the source of information for parents, about 39.2% (n=76) received from PHC doctors, followed by the Internet as a source at 30.4% (n=59).

**Table 4:** Parents' main concern about fever and their source of information. PHC: primary healthcare center.

Variable	Number	Percentage
Parents concerns about fever		
Convulsions	139	71.6
Dehydration	40	20.6
Brain damage	1	0.5
Hearing loss	8	4.1
Loss of consciousness	3	1.5
Source of information		
Relative/friends	18	9.3
Internet	59	30.4
TV	3	1.5
Written health materials	9	4.6
ER doctor	29	14.9
PHC doctor	76	39.2

Table 5 highlights that in the practice domain of parents towards feverish child, about 70.1% (n=136) applied cold compresses most of the time, followed by fever medicine administration at about 68.6% (n=132). Before visiting the doctor, about 33.5% (n=65) of parents administered more than one dose of fever medicine and only 3.6% (n=7) tried more than one fever medicine most of the time. Regarding difficulties faced by parents, about 28.4% (n=55) choose the right dose most of the time; on the other hand, only 14.9% (n=29) of parents do not have any difficulties.

**Table 5:** Practice status of parents towards feverish children in the study population.

Practice	Mostly (%)	Sometimes (%)	Never (%)	Total (%)
Apply cold compresses	136 (70.1)	55 (28.4)	3 (1.5)	194 (100)
Give fever medicine	133 (68.6)	41 (21.1)	20 (10.3)	194 (100)
Give plenty of fluids	103 (53.1)	63 (32.5)	28 (14.4)	194 (100)
Consult relatives or friends in the medical field	64 (33)	69 (35.6)	61 (31.4)	194 (100)
Consult relatives or friends	20 (10.3)	50 (25.8)	124 (63.9)	194 (100)
Take him immediately to the doctor	95 (49)	67 (34.5)	32 (16.5)	194 (100)
Give more than one dose before visiting the doctor	65 (33.5)	80 (41.2)	49 (25.3)	194 (100)
Try more than one fever medicine	7 (3.6)	52 (26.8)	135 (69.6)	194 (100)
Difficulties: choose the right medicine	43 (22.2)	56 (28.9)	95 (49)	194 (100)
Choose the right dose	55 (28.4)	56 (28.9)	83 (42.8)	194 (100)
Decide how frequent the doses	41 (21.1)	45 (23.2)	108 (55.7)	194 (100)
No difficulties	29 (14.9)	25 (12.9)	140 (72.2)	194 (100)

Table 6 mentions that about the health-seeking behavior of parents, nearly 88.1% (n=171) preferred to visit the doctor when their child had a high fever and 87.1% (n=169) of parents when there is no response to fever medicine. Only 35.1% (n=68) of parents preferred to visit the doctor irrespective of the fever level.

**Table 6:** Health-seeking behavior of parents towards their feverish child.

Health-seeking behavior	Yes (%)	No (%)	Total (%)
If a child develops a fever, regardless of its level	68 (35.1)	126 (64.9)	194 (100)
If a child has a high fever	171 (88.1)	23 (11.9)	194 (100)
If no response to fever medicine	169 (87.1)	25 (12.9)	194 (100)
In no improvement after 24 hours	132 (68)	62 (32)	194 (100)
If no clear reason (diarrhea, teething,...)	133 (68.6)	61 (31.4)	194 (100)
If a child has other symptoms (rhinorrhea, cough, ear pain, diarrhea,...)	151 (77.8)	43 (22.2)	194 (100)
If a child’s age is less than three years	136 (70.1)	58 (29.9)	194 (100)

Table 7 states that no statistically significant association was observed between age group and gender with the source of information (P>0.05). Among the teachers, about 35.5% (n=22) received information from PHC doctors, while 38.6% (n=22) of housewives received information from PHC doctors. Among individuals in other occupations, 42.7% (n=32) received information from PHC doctors. A statistically significant association was observed between occupation categories in relation to source of information (P<0.05).

**Table 7:** Association of demographic factors with the source of information of the participants. PHC: primary healthcare center. ^*^Statistically significant.

Variable	Relatives/friends (%)	Internet (%)	TV (%)	Written health materials (%)	ER doctor (%)	PHC doctor (%)	Total (%)
≤45 years	10 (7.9%)	36 (28.6%)	1 (0.8%)	5 (4.0%)	24 (19.0%)	50 (39.7%)	126 (100%)
>45 years	8 (11.8%)	23 (33.8%)	2 (2.9%)	4 (5.9%)	5 (7.4%)	26 (38.2%)	68 (100%)
X^2^=6.828, 5 df, P=0.234							
Male	8 (6.9%)	35 (30.2%)	3 (2.6%)	5 (4.3%)	20 (17.2%)	45 (38.8%)	116 (100%)
Female	10 (12.8%)	24 (30.8%)	0 (0.0%)	4 (5.1%)	9 (11.5%)	31 (39.7%)	78 (100%)
X^2^=4.879, 5 df, P=0.431							
Teachers	0 (0.0%)	22 (35.5%)	0 (0.0%)	1 (1.6%)	17 (27.4%)	22 (35.5%)	62 (100%)
Housewife	9 (15.8%)	16 (28.1%)	0 (0.0%)	2 (3.5%)	8 (14.0%)	22 (38.6%)	57 (100%)
Others (police, military,...)	9 (12.0%)	21 (28.0%)	3 (4.0%)	6 (8.0%)	4 (5.3%)	32 (42.7%)	75 (100%)
X^2^=29.268, 10 df, *P=0.001							

## Discussion

The present cross-sectional study was conducted with the objective of assessing parent knowledge and practice towards feverish child during the period from March 2023 to March 2024. Fever is a very common symptom among the pediatric age group and could be due to respiratory, gastrointestinal, and many other infective conditions of origin. Parents' adequate knowledge and practice about feverish child can lead to positive outcomes and reduce complications following fever.

In our study, 82% of participants were above the age of 35, and in contrast to our finding, only 56% within the same age group were observed by a study conducted in Riyadh [14]. About 40.2% of females participated in our study; almost the same observation of 44.3% of females participated in an Indian study [16]. More number of female (77.4%) participants were observed in the pediatric clinics of Unaizah, and this high percentage could be due to a different study setting [15].

About 69.6% of parents stated that fever is harmful and 29.4% mentioned very harmful. A study conducted in Riyadh stated that about 95% of parents mentioned fever as harmful [14]. Studies from different countries represented the prevalence range of fever as harmful from 60% to 86%: in Ireland, a study mentioned 60.4% [17], a Nigerian study stated 82.7% [18], and a study in Taiwan revealed that 86.6% of parents consider fever to be harmful [19]. 

Concerning the education level of the participants, 62.4% of mothers and 72.7% of fathers had completed college-level education. On the other hand, local study conducted in Unaizah stated that 20.4% of parents had completed university education [15], and close to a similar observation in an Indian study, only 24.3% were graduated parents [16].

In a local study conducted in Riyadh, 62.9% of the participants had an income level of more than 2400$ per month, consistent with our finding of 62.4% [14]. On the contrary, an international study conducted in Malaysia found that only 4.5% had an income level of more than 1700$ per month [20]. Differences in economic status varies from countries to countries.

Regarding measuring temperature, about 87.1% of parents measure their child's temperature by touching the forehead. In Morocco, a published study revealed that 44.5% of parents measure their child's temperature in the same way [12], a finding similar to local study where 43.3% of parents did the same [15]. About 41.2% of parents brought their child to a PHC or ER when their child has a fever. Another study stated that 60% of parents take their child to health center, hospital, or ER [14].

The majority (71.6%) of the parents in our study were concerned about convulsions, a similar finding of the same result observed in multiple local and international published studies, which reported convulsions as the main concern but with different percentages. Regarding local studies, one conducted in Riyadh found that 74% of parents chose convulsions as their main concern [14], while another in Unaizah reported 79.7% [15]. In Germany and India, 56.3% [21] and 44.3% [16] of parents, respectively, reported the same concern. The second concern was dehydration, reported by about 20.6%. An Australian study indicated that 63.6% of parents considered dehydration a concern [22], a finding supported by a 2017 published Iranian study [23].

Regarding the source of information for parents, about 39.2% were received from PHC doctors. Similar results were seen in a study published by Crocetti et al., where 46% of parents reported the same source [5]. One local study done by AlAteeq et al. mentioned that 39.2% of parents consider pediatricians as their primary source of information [14]. In our study, the Internet was the first source of information for 30.4% of parents, whereas an Iranian study revealed that only 10.9% of parents used media as a source of information [24]. The difference could be due to a knowledge gap as well as the attitude of parents towards obtaining information. 

Regarding parents' practice towards their feverish child, about 70.1% applied cold compresses most of the time. Close to our findings, a local study revealed that 78.4% of parents applied cold sponging to treat the fever [25]. A similar observation was found in a Moroccan study, which stated that towels soaked with cold water were commonly applied when their child had a fever [12]. A study conducted by Fallah Tafti et al. revealed that when the child had a fever, mothers mostly practiced foot baths with cold water, and they concluded that foot baths reduce the fever [23]. In most countries, parents practice non-pharmacological therapy as the first choice for controlling fever.

In our study, fever medicine administration was about 68.6%, in France study reported high percentage of 91% of parents administered fever medication before bringing their child to the health care professionals [26]. On the other hand Turkish study mentioned only 42.4% of parents administered fever medication to their child [27]. Low percentage of medication administration to the child could be the assumption of unnecessary medication to the child.

Nearly 88.1% of parents preferred to visit the doctor when their child had a high fever, concerning health-seeking behavior in the current study. A slightly lower percentage, 74.4% of parents consulted the doctor when their child had a high fever [14]. Another study conducted in Riyadh stated that about 83.3% of participants called the doctor when their child had a fever higher than 380°C [25]. Close to 70% of parents visited general practitioners because of their feverish child in an Ireland study [17]. The high percentage of health-seeking behavior could be due to increased awareness among parents about fever.

Some of the limitations observed in our study include misunderstandings of the questions in the self-administered questionnaire, difficulties in writing some answers, and the use of a convenience sampling method. The strength of this study is that it successfully encouraged parents to participate at the crowded PHCs.

## Conclusions

In conclusion, nearly all participants identified fever as harmful to their children. However, poor knowledge was observed for defining normal body temperature. There was a statistically significant association observed between the different occupations of the participants and source of information. In relation to health-seeking behavior practice, nearly two-thirds of parents had good practice.
